# Role of 20-Hydroxyeicosatetraenoic Acid in Mediating Hypertension in Response to Chronic Renal Medullary Endothelin Type B Receptor Blockade

**DOI:** 10.1371/journal.pone.0026063

**Published:** 2011-10-07

**Authors:** Joshua S. Speed, Eric M. George, Marietta Arany, Kathy Cockrell, Joey P. Granger

**Affiliations:** 1 Department of Physiology and Biophysics, University of Mississippi Medical Center, Jackson, Mississippi, United States of America; 2 Center for Excellence in Cardiovascular–Renal Research, University of Mississippi Medical Center, Jackson, Mississippi, United States of America; INSERM, France

## Abstract

**Background:**

The renal medullary endothelin (ET-1) system plays an important role in the control of sodium excretion and arterial pressure (AP) through the activation of renal medullary ET-B receptors. We have previously shown that blockade of endothelin type B receptors (ET-B) leads to salt-sensitive hypertension through mechanisms that are not fully understood. One possible mechanism is through a reduction in renal medullary production of 20-hydroxyeicosatetraenoic acid (20-HETE). 20-HETE, a metabolite of arachidonic acid, has natriuretic properties similar to ET-B activation. While these findings suggest a possible interaction between ET-B receptor activation and 20-HETE production, it is unknown whether blockade of medullary ET-B receptors in rats maintained on a high sodium intake leads to reductions in 20-HETE production.

**Methodology/Principal Findings:**

The effect of increasing sodium intake from low (NS = .8%) to high (HS = 8%) on renal medullary production of 20-HETE in the presence and absence of renal medullary ET-B receptor antagonism was examined. Renal medullary blockade of ET-B receptors resulted in salt sensitive hypertension. In control rats, blood pressure rose from 112.8±2.4 mmHg (NS) to 120.7±9.3 mmHg (HS). In contrast, when treated with an ET-B receptor blocker, blood pressure was significantly elevated from 123.7±3.2 (NS) to 164.2±7.1 (HS). Furthermore, increasing sodium intake was associated with elevated medullary 20-HETE (5.6±.8 in NS vs. 14.3±3.7 pg/mg in HS), an effect that was completely abolished by renal medullary ET-B receptor blockade (4.9±.8 for NS and 4.5±.6 pg/mg for HS). Finally, the hypertensive response to intramedullary ET-B receptor blockade was blunted in rats pretreated with a specific 20-HETE synthesis inhibitor.

**Conclusion:**

These data suggest that increases in renal medullary production of 20-HETE associated with elevating salt intake may be, in part, due to ET-B receptor activation within the renal medulla.

## Introduction

Endothelin (ET-1) was first isolated and characterized in 1988 as a very potent vasoconstrictor produced by vascular endothelial cells [1]. Two receptor subtypes were later identified: ET-A and ET-B. ET-A receptors are responsible for the vasoconstrictor properties of ET-1, and chronic activation results in hypertension [2]. Their role in blood pressure regulation has been extensively researched. In contrast, ET-B receptors are located on the vascular endothelium and activation results in vasodilation; however, renal ET-B receptors have been found to be important in many facets of renal function including renal blood flow and electrolyte transport [3]. In fact, the renal medulla produces more ET-1 than any other site in the body [4], and activation of ET-B receptors located here causes natriuresis through a reduction in Na^+^ reabsorption in the collecting duct and thick ascending loop of Henle [5], [6], [7]. Furthermore, several studies indicate that renal medullary endothelin is important in the maintenance of fluid and electrolyte homeostasis, and this system becomes increasingly important as Na^+^ intake is elevated [8], [9]. Moreover, a reduction in renal production of ET-1 may be important in the pathogenesis of essential salt sensitive hypertension [10], [11], however the mechanisms by which renal medullary ET-1 enhances pressure natriuresis have yet to be fully elucidated.

One important mechanism of ET-1 induced natriuresis is through increases in nitric oxide (NO) production. For instance, increasing dietary Na^+^ enhances eNOS expression in the medullary thick ascending loop of Henle, however this is attenuated by ET receptor blockade [6]. Furthermore, knockout of ET-1 production by the collecting duct results in salt sensitive hypertension associated with reductions in urinary nitrate/nitrite and renal medullary expression of nitric oxide synthase (NOS) I and III [12]. Finally, the acute, natriuretic response to intramedullary ET-B activation can be attenuated by a NOSI inhibitor [13]. While substantial evidence implicates NO in mediating the renal effects of ET-1, growing evidence suggest that 20-Hydroxyeicosatetraenoic Acid (20-HETE) may also play an important role.

20-HETE is a metabolite of arachidonic acid metabolism though the cytochrome p-450 pathway, specifically the CYP4A family in rats and CYP4F in humans. 20-HETE has actions similar to those of ET-1 both in the vasculature and the renal medulla [14]. Chronic blockade of 20-HETE production results in salt sensitive hypertension [15], [16], as does chronic, systemic ET-B blockade [8]. Within the kidney, both 20-HETE and ET-B receptor activation inhibit Na^+^ reabsorption by the proximal tubule and the medullary thick ascending loop of Henle [6], [17], [18]. While chronic ET-B blockade is associated with a reduction in renal medullary CYP4A protein expression [19], the functional significance of this interaction in the control of blood pressure has yet to be determined. Therefore, the specific goal of our study was to determine if chronic increases in salt intake lead to enhanced formation of 20-HETE by the renal medulla and to determine if this relationship is altered during chronic intramedullary infusion of an ET-B antagonist. Finally, we wanted to determine if the increase in blood pressure in response to chronic intramedullary ET-B blockade is blunted when 20-HETE production is inhibited.

## Results

**Figure 1:**
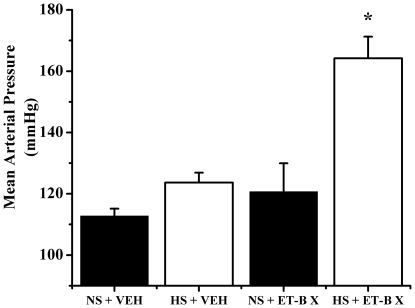
Mean arterial pressure in response to chronic intramedullary blockade of ET-B receptors. Chronic intramedullary blockade of ET-B receptors causes a slight increase in MAP in rats on a normal salt diet (112.8±2.4 in vehicle vs. 120.7±9.3). However, rats placed on high salt diet had a much greater elevation in MAP in response to IM ET-B blockade (123.7±3.2 vs. 164.2±7.1). * denotes p<.05 vs. all other groups.

**Figure 2:**
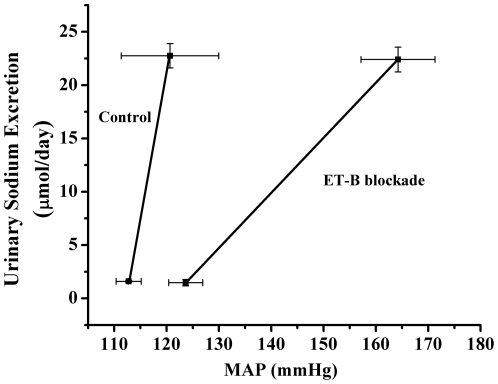
Pressure natriuresis relationship in response to intramedullary ET-B blockade. In response to chronic intramedullary ET-B blockade, there is a rightward shift of the pressure natriuresis relationship suggesting there is an impairment of the kidneys' normal ability to excrete salt and water.

In order to determine if chronic intramedullary blockade of ET-B receptors causes a reduction in pressure natriuresis and salt sensitive hypertension, we examined the sodium excretion and blood pressure relationship in rats treated with an ET-B receptor antagonist, specifically delivered to the renal medulla. Our data indicates that in response to intramedullary blockade, rats on a normal salt diet had only a slight elevation in blood pressure; however, rats placed on a high salt diet had a significant elevation in pressure (Figure 1). We also indicate that under steady state conditions, the pressure natriuresis relationship is shifted rightward, with a reduction in slope (Figure 2), in response to chronic intramedullary blockade of ET-B receptors, suggesting a reduction in the kidneys’ ability to excrete salt and water.

**Figure 3:**
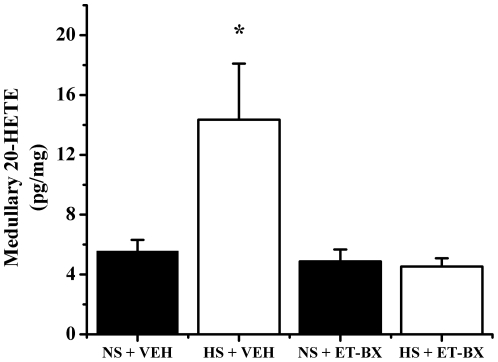
Renal medullary tissue levels of 20-HETE in response to blockade of medullary ET-B receptors. In response to increasing salt intake in male SD rats, renal medullary 20-HETE levels are significantly elevated (5.6±0.75 vs. 14.3±3.7, n = 4 and n = 7 respectively). With chronic intramedullary blockade of ET-B receptors, this response is completely abolished (4.9±0.79 vs. 4.5±0.55, n = 5 and n = 6 respectively). * denotes p<.05 vs. NS + VEH.

**Figure 4:**
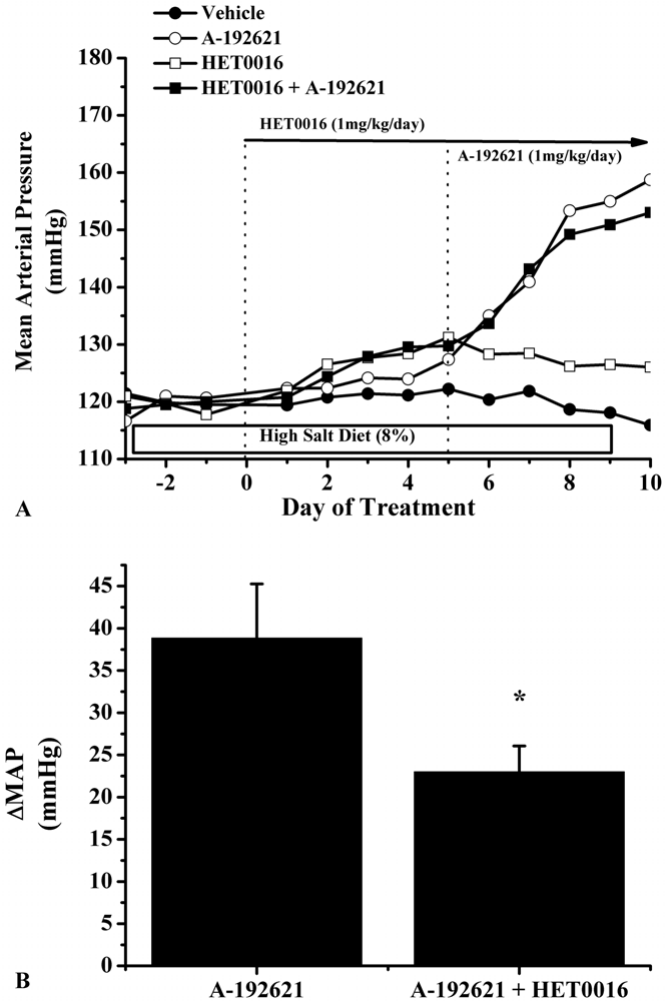
Mean arterial pressure in rats with intramedullary blockade of ET-B receptors in the presence and absence of a 20-HETE inhibitor. **A**) This figure illustrates the changes in blood pressure in response to chronic intramedullary infusion of the ET-B antagonist, A-192621, the 20-HETE inhibitor, HET0016, and rats pretreated with HET0016, and then administered A-192621. **B**) Illustrates that the increase in blood pressure in response to intramedullary blockade of ET-B receptors is blunted when the rats are pretreated with a 20-HETE inhibitor. *indicates that p<0.05 vs. A-192621 treated group.

Our data also indicates that rats placed on a high salt diet have a significant increase in renal medullary 20-HETE levels. When treated with an ET-B receptor blocker, this increase is completely abolished (Figure 3). Our data shows that only part of the blood pressure effects of ET-B receptor blockade is related to 20-HETE. In fact when we blocked 20-HETE production alone we report that blood pressure increased by approximately 10 mmHg and when we blocked the ET-B receptor in the presence of a 20-HETE inhibitor, the blood pressure response was about 10–12 mmHg lower than the group of rats receiving 20-HETE inhibitor alone. Thus, the blood pressure effect of the ET-B receptor blockade is attenuated when 20-HETE levels are clamped by a 20-HETE inhibitor. (Figure 4)

## Discussion

Previous studies indicate that ET-B receptors are important in the renal response to an increase in salt intake, and loss of function of these receptors results in hypertension [8], [20]. While blockade of ET-B receptors throughout the entire body causes salt-sensitive hypertension, the importance of renal medullary ET-B receptors in mediating the chronic blood pressure response should be considered. Our data indicates that the hypertension caused by ET-B receptor blockade is very closely associated with renal medullary ET-B receptors. As shown in Figure 1, chronic blockade of ET-B receptors, specifically in the renal medulla, results in hypertension very similar to that seen in systemic ET-B blockade [8]. This is a consequence of a reduction in the kidneys’ ability to excrete salt and water as evidenced by the rightward shift and reduced slope of the pressure natriuresis relationship after 7 days of ET-B blockade (Figure 2). Therefore, we can conclude that activation of renal medullary ET-B receptors is a necessary response to increasing salt intake and is important in the maintenance of water and electrolyte homeostasis.

We next wanted to determine if there is an important interaction between endothelin and 20-HETE in the regulation of blood pressure during chronic elevations in salt intake. It is well established that multiple components of the ET-1 system are upregulated within the renal medulla in response to increasing salt intake [21], and this contributes to the kidneys’ function in maintaining salt homeostasis and blood pressure. Because it has become increasingly apparent that 20-HETE has natriuretic properties, we hypothesized that increasing salt intake would lead to an elevation in renal medullary production of 20-HETE, and that this response would be attenuated with chronic intramedullary ET-B blockade. Indeed, rats placed on a HS diet had almost 3-fold higher renal medullary tissue levels of 20-HETE than rats on a NS diet. This response was completely abolished in rats with chronic intramedullary ET-B blockade (Figure 3) suggesting that increases in 20-HETE production in response to high salt is a result of ET-B receptor activation.

Since we saw a significant reduction in 20-HETE production in rats chronically treated with an ET-B receptor antagonist, we next wanted to test the functional significance of this observation by comparing the hypertensive response to intramedullary ET-B receptor antagonism in the presence and absence of a 20-HETE inhibitor. We proposed that if 20-HETE plays an important role in mediating the chronic effects of renal medullary ET-1 on pressure natriuresis and blood pressure regulation during increases in sodium intake, the blood pressure response to medullary ET-B receptor blockade would be attenuated in the presence of a 20-HETE inhibitor. In the present study, we found that chronic intramedullary infusion of the ET-B receptor antagonist increased blood pressure by 40 mmHg. In contrast, intramedullary infusion of a 20-HETE inhibitor alone increased blood pressure by only 10 mmHg, thus, the maximum role for 20-HETE in mediating the blood pressure response to ET-B receptor blockade is about 10 mmHg. Consistent with this is our finding that in the presence of a 20-HETE inhibitor, chronic intramedullary infusion of the ET-B receptor antagonist increased blood pressure by only 25 mmHg or about 15 mmHg lower than when we infused ET-B receptor antagonist alone into the renal medulla. While these data suggest that 20-HETE may, in part, play an important role in mediating ET-B receptor function in response to a high salt intake, we cannot rule out an important interaction between ET-B receptor blockade, 20-HETE, and nitric oxide. As pointed out in the introduction, there is substantial evidence implicating NO in mediating some of the renal effects of ET-1, especially the collecting duct actions of ET-1. However, our data also implicates an important interaction between 20-HETE and ET-1, an interaction that may occur at nephron sites such as the medullary thick ascending limb where 20-HETE is produced.

In conclusion, we have found that renal medullary production of 20-HETE is elevated in response to a high salt intake, and this response is completely abolished in rats treated with an ET-B receptor antagonist directly in the renal medulla. Finally, we found that the elevation in blood pressure in response to intramedullary ET-B receptor blockade was significantly blunted in the presence of a 20-HETE inhibitor, suggesting that 20-HETE may play a role in mediating the natriuretic effects of ET-1 during a high salt intake. Therefore, in response to a high salt diet, renal medullary production of endothelin-1 is enhanced, thus activating mainly ET-B receptors located on the medullary thick ascending loop of Henle and collecting ducts. When activated, it is believed that production of NO and 20-HETE lead to a reduction in Na uptake by these tubule segments and, ultimately, an increase in Na^+^ excretion, therefore, providing a mechanism for the kidney to increase Na^+^ and water excretion, without elevations in blood pressure.

## Materials and Methods

### Ethics Statement

All studies were performed using age matched Sprague Dawley rats purchased from Harlan, Inc. (Indianapolis, IN). Animals were housed in a temperature-controlled room (23°C) with a 12∶12 hour light/dark cycle. All surgeries were performed under isoflurane anesthesia. All experimental procedures executed in this study were in accordance with National Institutes of Health guidelines for use and care of animals and approved by the Institutional Animal Care and Use Committee (IACUC) at UMMC (Protocol number 0258D)

### Drugs

For specific intramedullary blockade of the ET-B receptor, 1 mg/kg/day of A-192621 was used. This dose was based on the ED_50_ observed in systemic administration of the drug [22], as well as unpublished data from our laboratory in which we found that intravenous infusion of 1 mg/kg/day had minimal effects on blood pressure. For blockade of 20-HETE production, HET0016 was used at a dose of 1 mg/kg. This dose was determined from previous reports that IV infusion of HET0016 at 10 mg/kg significantly reduced urinary levels of 20-HETE, but not EET's, which would suggest that HET0016 is a highly specific inhibitor of CYP4A proteins [15]. This dose was then extrapolated to fit regional blood flow of the kidney.

### Experiment 1: Effect of Na^+^ intake on renal medullary 20-HETE production and the effect of ET-B blockade on this relationship

#### Experimental protocol

To examine the long-term interaction between ET-1 and 20-HETE during chronic increases in dietary sodium intake, we first determined if renal medullary production of 20-HETE is elevated in response to increases in dietary sodium intake in normal SD rats. Then we examined the effect of increases in dietary sodium intake on renal medullary production of 20-HETE during chronic ET-B receptor blockade. Male SD rats (300–350 g) were randomly distributed among 4 groups including a control on Normal Sodium (NS, 0.8% NaCl) diet (n = 4), control on High sodium diet (HS, 8% NaCl) (n = 7), Endothelin type B receptor (ET-B) blockade on NS (n = 5), and ET-B receptor blockade on HS (n = 6). Next, one kidney was removed and the other was instrumented with a chronic indwelling catheter placed into the interstitium of the renal medulla. The catheters were made of V-1 tubing connected to V-3 tubing with superglue. A round piece of alliedsil (Allied Biomedical, Paso Robles, CA) sheeting was placed between the two portions in order to secure the catheter to the kidney. The V-1 portion was inserted 4–5 mm into the kidney and secured to the renal capsule with vetbond. The rats were given 7 days to recover and instrumented with telemetry probes (Data Science International, St. Paul, MN) for 24-hour collection of blood pressure and placed on respective diets. After pressure normalized post surgery, the rats were tethered, placed in metabolic cages, and infusion of vehicle (70% ethanol, 0.6 µl/min) to the medullary interstitium was began. Control pressure was taken for 3 days and urine was collected on ice for 24 hours on the final day for measurement of 20-HETE. Next, vehicle or the ET-B antagonist A-192621(Abbott Laboratories, Abbott Park, IL) (1 mg/kg/day) was infused for seven days, and urine was collected on the final day. The rats were euthanized and tissues were harvested for measurements of 20-HETE production. Kidneys were dissected to ensure proper placement. Urinary Na^+^ concentration was measured using an EasyLyte Na^+^/K^+^ analyzer (Medica Corp.), and Na^+^ excretion was calculated by multiplying the urinary Na^+^ concentration by the urine flow per 24 hours.

#### Measurement of 20-HETE

Lipids were separated from renal homogenates using ≈50 mg of tissue by the following protocol. First, samples were homogenized in a.1 M potassium phosphate buffer (.1 M monobasic KPO, 1 M dibasic KPO, 250 mM sucrose, 1 mM ETDA, and 1 µL/mL PMSF). The samples were acidified with 1 M formic acid to bring the pH between 3.5 and 4.0. 2 ng of 20-hydroxyeicosa-6(Z), 15(Z)-dienoic acid was added to each sample as an internal standard to account for loss during extraction. Next, three mL of ethyl acetate were added and the samples were vortexed for 2 minutes. They were then centrifuged for 3 minutes at 2200 rpm. The upper organic layer was transferred to another glass tube and 1 mL of distilled water was added to the samples, and they were vortexed again for 2 minutes. Once again, the upper organic layer was transferred to another glass vial and the ethyl acetate was evaporated under nitrogen until samples were dry. They were quickly capped and stored at −80°C until assayed by mass spectrometry. 20-HETE was measured by liquid chromatography mass spectrometry (LCMS) as previously described [23].

### Experiment 2: Functional significance of 20-HETE in chronic, intramedullary ET-B blockade

To determine the role of 20-HETE in mediating the long-term blood pressure response to chronic ET-B receptor blockade, we examined the effect of IM blockade of 20-HETE production on the blood pressure response to chronic ET-B receptor blockade. Male SD rats were uninephrectomized and chronic renal medullary interstitial catheter was placed in the opposite kidney, as previously described. The rats were then instrumented with telemetry probes (Data Science International, St. Paul, MN) for 24 hr measurement of blood pressure. The rats were then placed on a high Na^+^ diet and allowed to heal for one week. Baseline pressure was measured for 3 days. Next, a 20-HETE inhibitor (HET0016, 1 mg/kg/day) was administered IM for five days. The rats were then placed in metabolism cages on the last two days for urine collection. Finally, an ET-B antagonist (A-192621, 1 mg/kg/day IM) was administered via IM infusion for five days and rats were placed in metabolic cages on the final day for 24 hour urine collection. Blood pressure was monitored daily and recorded as 24 hour average. The doses used were extrapolations from systemic blockade studies based on regional blood flow [8], [15].

### Statistical Analyses

All data is presented as mean ± standard error of the mean. In experiment 1, blood pressure was averaged over the last 3 days of chronic intramedullary infusion of A-192621. Blood pressure averages and 20-HETE data were analyzed by one-way analysis of variance, and Tukey’s post hoc test was used for comparison between groups. In experiment 2 (Figure 4), the change in blood pressure between groups was analyzed by Student’s t-test.
